# Photocatalytic degradation of methylene blue with spent FCC catalyst loaded with ferric oxide and titanium dioxide

**DOI:** 10.1038/s41598-020-69643-2

**Published:** 2020-07-29

**Authors:** Jiasheng Xu, Te Zhang, Jie Zhang

**Affiliations:** 1 College of Chemistry, Chemical Engineering and Environmental Engineering, Liaoning Shihua University, Fushun, 113001 People’s Republic of China; 2 Liaoning Province Key Laboratory for Synthesis and Application of Functional Compounds, College of Chemistry and Chemical Engineering, Bohai University, Jinzhou, 121013 People’s Republic of China

**Keywords:** Materials chemistry, Photocatalysis

## Abstract

The spent fluid catalytic cracking (FCC) catalyst has been loaded with ferric oxide (Fe_2_O_3_) and titanium dioxide (TiO_2_). Fe-Ti/SF composite (loaded with 5 wt% TiO_2_ and 5 wt% Fe_2_O_3_), Fe/SF composite (loaded with10 wt% Fe_2_O_3_) and Ti/SF composite (loaded with 10 wt% TiO_2_) have been fabricated via a modified-impregnation method. The band gaps of the Fe-Ti/SF, Fe/SF and Ti/SF composites (evaluated by the energy versus [F(R∞)*hv*]^n^) are 2.23, 1.98 and 3.0 eV, respectively. Electrochemical impedance spectroscopy shows that the Fe-Ti/SF has lower electron transfer resistance, it has the small charge transfer resistance and fast charge transfer rate. The interparticle electrons transfer between the Fe_2_O_3_ and TiO_2_, which can improve the separation of the photo-electrons and holes. The holes transfer from valence band of TiO_2_ to the valence band of Fe_2_O_3_, which can provide more active sites around the adsorbed molecules. The methylene blue degradation efficiencies (with the Fe-Ti/SF, Fe/SF and Ti/SF composites) are ~ 94.2%, ~ 22.3% and ~ 54.0% in 120 min, respectively. This work reveals that the spent FCC catalyst as supporter can be loaded with Fe_2_O_3_ and TiO_2_. This composite is highly suitable for degradation of methylene blue, which can provide a potential method to dispose the spent FCC catalyst in industry.

## Introduction

In the oil refinery, the fluid catalytic cracking (FCC) is an important secondary conversion procrss^1–4^. The crude oil can be converted into the valuable small molecules products, which is an essential process for gasoline production^5^. In the FCC process, the catalytic activity of FCC catalyst decreases after several cycles. Metals (V, Ni, and Fe) accumulation occurs via deposition and incorporation into the FCC catalyst body^6,7^. There are about 840,000 t spent FCC catalyst consumed in the world every year and it is anticipated annual increase of 5%^8,9^. The spent FCC catalyst are mainly treated via landfill^10^. Some researcher explore the coating of spent FCC catalyst as anticorrosive and antimicrobial material, other researcher uses the spent FCC catalyst to recover the precious metal and rare earth, and use spent FCC catalyst as admixtures in mortar and concrete production^11–13^. Zeolite Y is the main components of the FCC catalyst^14^. When the FCC catalyst is deactivated, the pore volume of catalyst is almost intact, which still can be used as a carrier^15^.

Titanium dioxide (TiO_2_) has been extensively investigated for photocatalytic reaction due to its chemical stability and lack of toxicity^16–19^. TiO_2_ is a wide band gap semiconductor material (3.2 eV)^20–23^. In photocatalytic reaction, the charge carrier of the TiO_2_ is fast recombinated, which can be prevented effectively by the heterogeneous structures (with the narrow band gap semiconductor materials)^24,25^. Fe_2_O_3_ with narrow band gap (2.2 eV) tends to have short carrier diffusion lengths. Therefore, Fe_2_O_3_ extends the optical absorption edge to visible light region for TiO_2_. Eskandari et al. and Davari et al. synthesized TiO_2_/Fe_2_O_3_/zeolite composite, which displayed 89% and 80% photocatalytic degradation efficiency^26–29^.

In this work, spent FCC catalyst loaded with Fe_2_O_3_ and TiO_2_ was fabricated via a modified-impregnation method. The photocatalytic performance the Fe-Ti/SF, Fe/SF and Ti/SF samples are evaluated by the degradation efficiency of the methylene blue. The recycling experiments of the Fe-Ti/SF composite are implemented by the methylene blue degradation, which evaluates the stability of the Fe-Ti/SF composite. The advantage of this work is that it provides a new way to treat spent FCC catalyst. At present, the spent FCC catalyst are mainly treated by landfill. This method caused severe land pollution and polluted groundwater. The novelty of this work is the introduction spent FCC catalyst as supporter. These composites fabricated via this modified-impregnation method can provide an effective route to dispose the spent FCC catalyst in industry.

## Materials and methods

### Fabrication of the composites

In a typical experimental procedure, the spent FCC catalyst was loaded with titanium dioxide (5 wt% TiO_2_) and ferric oxide (5 wt% Fe_2_O_3_), it was prepared as follows. 0.85 mL tetrabutyl titanate and 0.31 g ammonium ferric oxalate were dissolved into 4 mL of H_2_O_2_ solution (~ 30%). Then 4 g spent FCC catalyst was added into this solution. Finally, this mixture was grinded for 2 h and calcined at 800 °C for 4 h. This sample is denoted as Fe-Ti/SF composite. The other two samples, i.e. Fe/SF composite, the spent FCC catalyst was loaded with ferric oxide (10 wt% Fe_2_O_3_) and Ti/SF composite, the FCC catalyst was loaded with titanium dioxide (10 wt% TiO_2_) were obtained in the above experimental procedure.

### Characterizations

The structures of the fabricated composites were tested by power XRD (Rigaku RAD-3C, Cu Kα radiation, 10° min^−1^, 2-*Theta* range 5°–80°). These composites morphology was analyzed by a Scanning Electron Microscope (SEM JEOL S-4800). Fourier transformation infrared (FT-IR) was tested using a spectrometer at a wavenumber covering the range of 400–4,000 cm^−1^. To measure band gap of these composites, UV–Vis light absorption was recorded on the Diffuse Reflectance spectrophotometer UV–vis. Nitrogen adsorption desorption tests were executed on a Micrometrics (Tristar 3000) instrument. Transmission electron microscope (TEM, JEOL LED JSM-6700F microscope, Japan) was used to investigate microstructure. Electrochemical impedance spectra (EIS) were tested via using a CHI660D workstation at room temperature. The electrochemical performances of the Fe-Ti/SF, Fe/SF and Ti/SF were analyzed. The working electrode were Fe-Ti/SF, Fe/SF and Ti/SF, respectively. The counter electrode was the Pt plate electrode and reference electrode were the calomel electrode. The electrolyte of the EIS measurements was the Na_2_SO_4_ aqueous solution (0.2 mol L^−1^).

### Photocatalytic tests

The adsorption and photocatalytic performance of the Fe-Ti/SF, Fe/SF and Ti/SF were tested by photocatalysis degradation of MB. In the experiment, 0.2 g of fabricated composite were added into 200 mL of methylene blue (10 ppm). The photocatalytic tests were driven by irradiation with the 300 W Xenon lamp for 120 min. Before the light irradiation, the reaction system was stirred for 40 min in darkness to ensure the adsorption–desorption equilibrium. The methylene blue was taken at given time interval (20 min). The solutions were centrifuged under 4,000 rpm before analysis.

## Results and discussion

**Figure 1:**
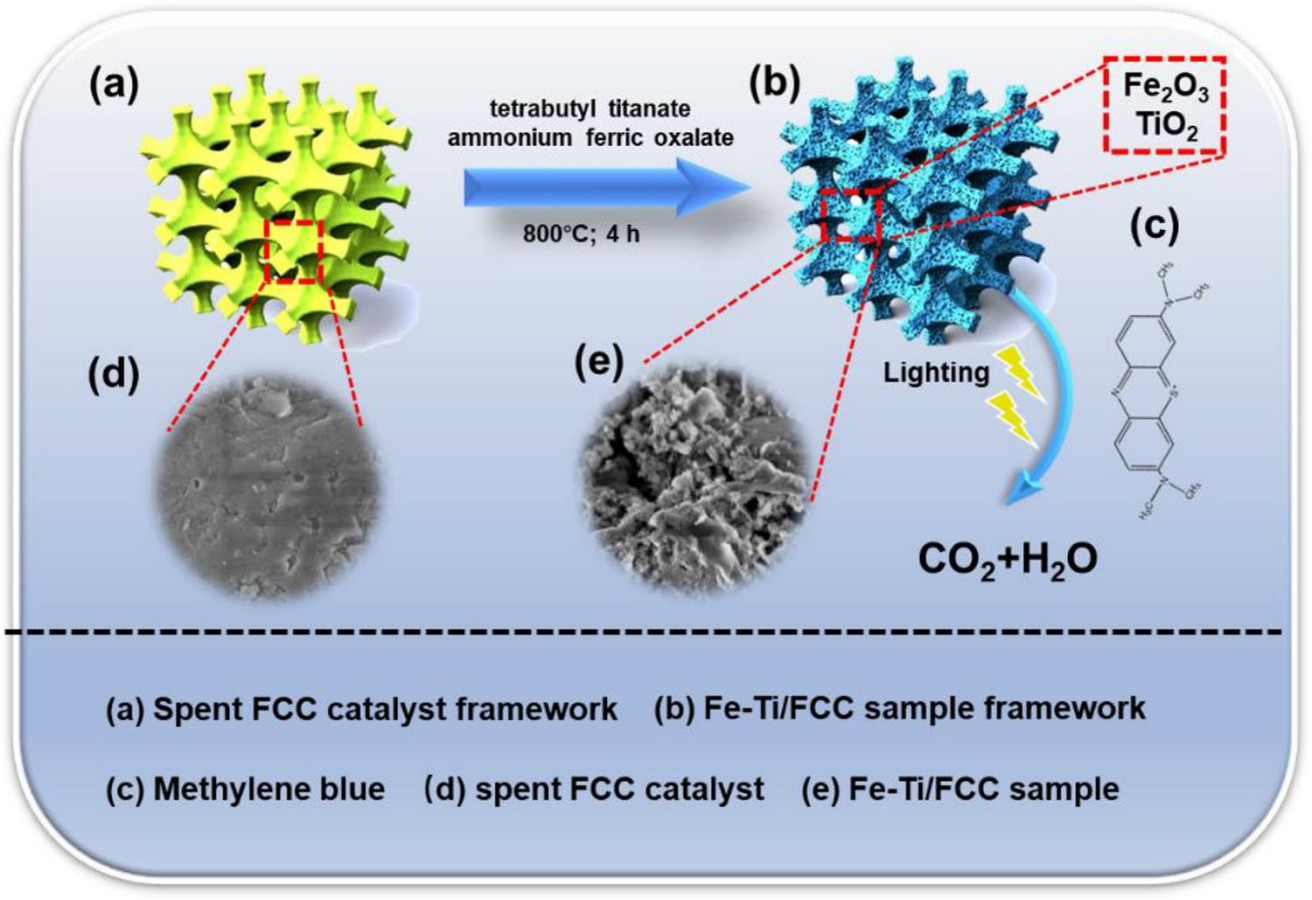
Schematic illustration for the Fe-Ti/SF composites fabricated process. (**a**) spent FCC catalyst framework, (**b**) Fe-Ti/SF framework, (**c**) methylene blue, (**d**) SEM images of spent FCC catalyst and (**e**) Fe-Ti/SF.

Figure 1 shows schematic illustration of the Fe-Ti/SF composite fabrication process. In a typical experimental procedure, 0.85 mL tetrabutyl titanate and 0.31 g ammonium ferric oxalate were dissolved into 4 mL of H_2_O_2_ solution (~ 30%). 0.85 mL tetrabutyl titanate and 0.31 g ammonium ferric oxalate were dissolved into 4 mL of H_2_O_2_ solution (~ 30%). Then 4 g spent FCC catalyst was added into this solution. Finally, this mixture was grinded for 2 h and calcined at 800 °C for 4 h. The degradation efficiency of methylene blue can be used to evaluate the photocatalytic performance of Fe-Ti/SF composite. The methylene blue can be degraded into the carbon dioxide (CO_2_) and water (H_2_O) at last.

**Figure 2:**
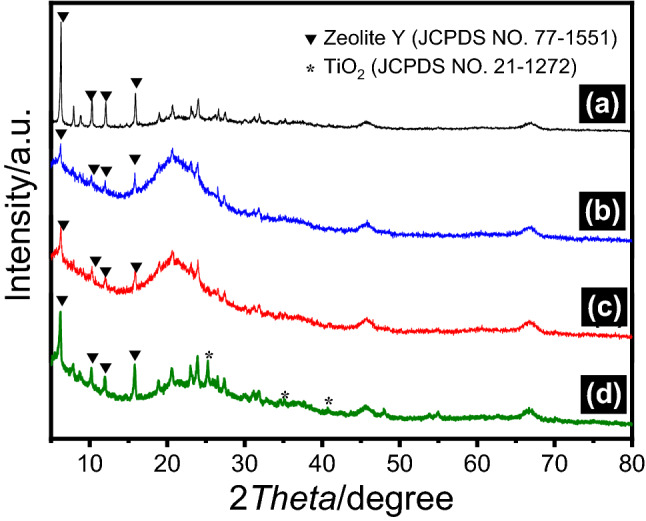
XRD patterns of these samples. (**a**) spent FCC catalyst, (**b**) Fe/SF, (**c**) Fe-Ti/SF and (**d**) Ti/SF.

XRD patterns of the spent FCC catalyst, Fe-Ti/SF, Fe/SF and Ti/SF are shown in Fig. 2 (2-*Theta* range from 5° to 80°). Figure 2a shows XRD patterns of the spent FCC catalyst. There are four diffraction peaks at 6.3°, 10.3°, 12.1° and 15.9°, which can be indexed to (111), (220), (311) and (331) planes of zeolite Y phase (JCPDS No. 77-1551), respectively. Figure 2b,c show the XRD patterns of the Fe/SF and Fe-Ti/SF composite. The intensity of the peak (111) in zeolite Y phase decreases after being loaded Fe_2_O_3_ or TiO_2_. In the range of 12°–25°, the peak intensity is higher than Fig. 2a. In Fig. 2d, there are three peaks at 26.4°, 35.3° and 40.1°. These peaks are corresponded to the (101), (004) and (200) planes of the TiO_2_ phase (JCPDS card No. 73-1764), respectively. The size of the loaded TiO_2_ is small, which causes the low diffraction peak. Compared with the zeolite Y (111), the diffraction peaks intensity of Fe_2_O_3_ is lower. Therefore, there no obvious diffraction peaks of Fe_2_O_3_.

**Figure 3:**
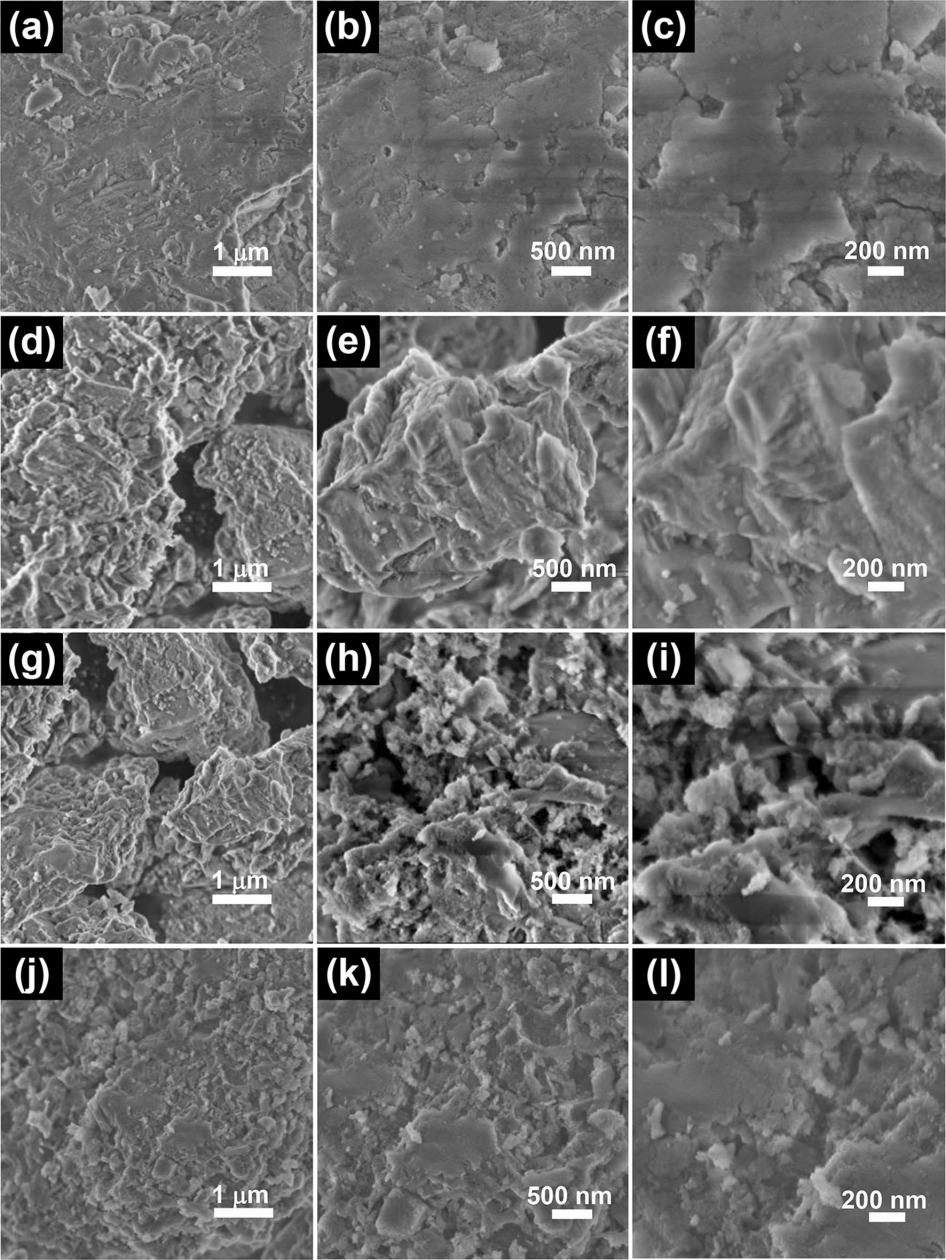
SEM images of the spent FCC catalyst, Fe-Ti/SF, Fe/SF and Ti/SF samples with different morphologies. (**a**–**c**) spent FCC, (**d**–**f**) Fe-Ti/SF, (**g**–**i**) Fe/SF, (**j**–**l**) Ti/SF.

Figure 3 presents scanning electron microcopy (SEM) images of the spent FCC catalyst, Fe/SF, Fe-Ti/SF and Ti/SF samples. In Fig. 3a–c, there are some cracks on the surface. In Fig. 3d, the surface of this composite is covered with Fe_2_O_3_. In Fig. 3e–f, the peripheral surface of the Fe/SF is coarse. Figure 3g–i show the Fe-Ti/SF composite, this composite is covered with Fe_2_O_3_ and TiO_2_. In Fig. 3i, because the TiO_2_ deposits in clusters of irregular shapes, the surface of Fe-Ti/SF composite is rough and jagged. Figure 3j–l show the morphologies of the Ti/SF composite. The TiO_2_ particles are loaded on the surface of supporter. As shown in Fig. 3l, the TiO_2_ particles with diameter raging from 50 to 200 nm. There is an aggregation phenomenon between TiO_2_. EDS mappings of the Fe-Ti/SF composite are shown in Supplementary Figure S1. It shows the corresponding mappings of Fe-Ti/SF composite, which clearly indicate the homogeneous of Si, O, Al, Fe and Ti elements. The ingredient of zeolite Y are Al_2_O_3_ and SiO_2_. The Al, Si and O elements originate from zeolite Y, the Fe and Ti are loaded on the zeolite Y.

XPS spectra are used to identify the valence states of the elements of the Fe-Ti/SF composite. Supplementary Figure S2 shows the XPS survey spectrum, which suggests the presence of Fe, Ti, Al, Si and O. In Supplementary Figure S2a, the peaks of Fe 2p_3/2_ and Fe 2p_1/2_ are overlap, the three binding energy peaks at 712.0, 721.6 and 725.7 eV. In Supplementary Figure S2b, two banding energy peaks at 458.7 and 464.3 eV correspond to the characteristic of the Ti 2p_3/2_ and Ti 2p_1/2_. In Supplementary Figure S2c, the peak of Al 2p is overlap, the banding energy peak at 74.6 eV. In Supplementary Figure S2d, the peak of Si 2p is overlap, the banding energy peak at 102.9 eV. In Supplementary Figure S2e, the binding energy peak at 531.8 eV correspond to the characteristic of the O 1 s.

**Figure 4:**
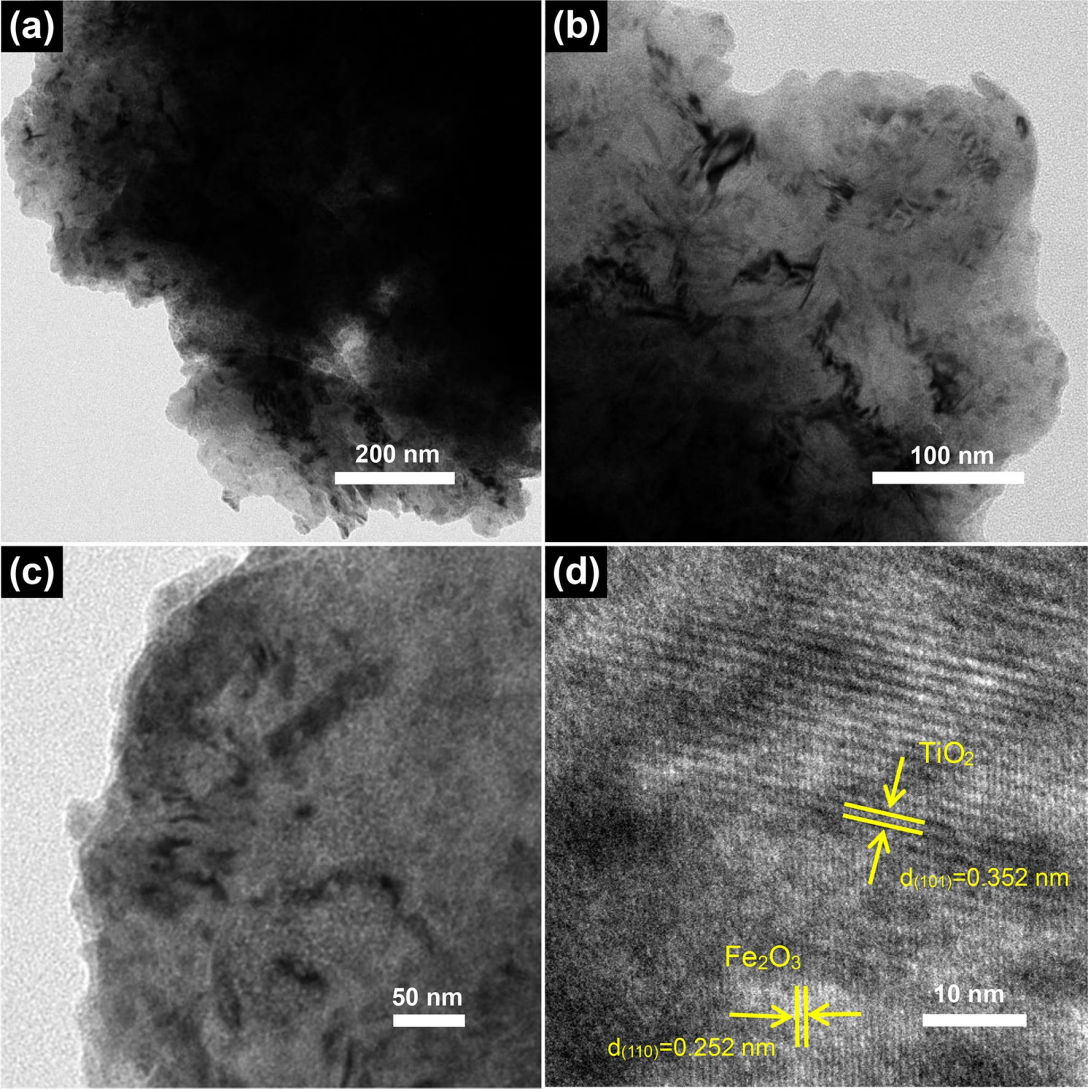
(**a**–**c**) TEM images of the Fe-Ti/SF, (**d**) HRTEM images of the Fe-Ti/SF.

Figure 4 shows the TEM images of the Fe-Ti/SF composite. In Fig. 4a–c, the Fe_2_O_3_ and TiO_2_ particles are attached to the surface of the spent FCC catalyst. The crystallography of the Fe-Ti/SF composite is investigated with high-resolution TEM (HRTEM). In the Fig. 4d, the nanostructured heterojunction is formed between Fe_2_O_3_ and TiO_2_. The intimately contacted interface is important for accelerating the separation of the electrons and holes. The HRTEM image in Fig. 4d shows two kinds of lattice fringes, the interplanar crystal spacing of 0.352 and 0.252 nm are corresponded TiO_2_ (101) and Fe_2_O_3_ (110), respectively. More TEM images of the Fe-Ti/SF composite are shown in Supplementary Figure S3a–c. The dark color regions under TEM indicate thickness of the zeolite Y. The TEM images further show that Fe_2_O_3_ and TiO_2_ are loaded on the surface of supporter.

**Figure 5:**
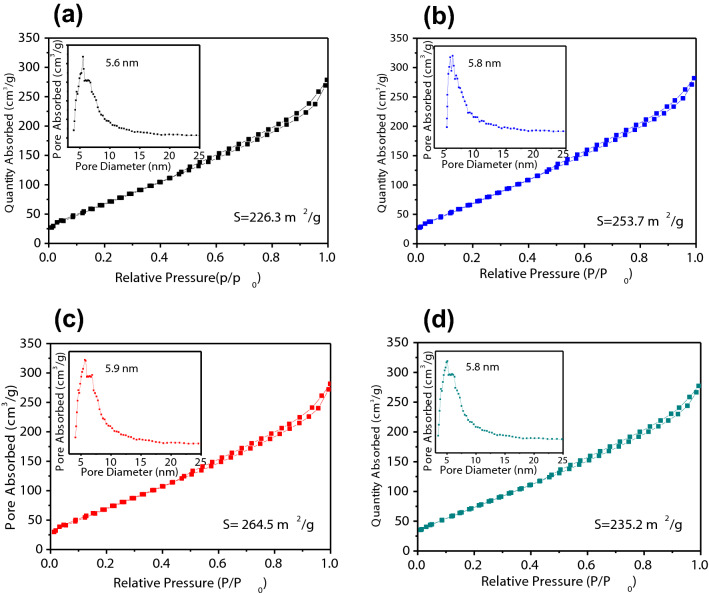
Brunauer–Emmett–Teller (BET) nitrogen adsorption and desorption isotherms of (**a**) spent FCC catalyst, (**b**) Fe/SF, (**c**) Fe-Ti/SF and (**d**) Ti/SF. The corresponding pore size distribution curves are illustrated in the inset.

In Fig. 5, the macropores properties of the spent FCC catalyst, Fe/SF, Fe-Ti/SF and Ti/SF are analyzed. These insets show the pore diameter of these samples. All the samples show the type-II isotherms, which are corresponded of macropores materials (IUPAC classification). The isotherms of these samples exhibit H3 hysteresis loops associated with the presence of macropores. The pore diameter of the spent FCC catalyst, Fe/SF, Fe-Ti/SF and Ti/SF are 5.6, 5.8, 5.9 and 5.8 nm, respectively. It shows that these samples are macroporous. The surface areas of these composites (spent FCC catalyst, Fe/SF, Fe-Ti/SF and Ti/SF) are calculated to be 226.3, 253.7, 264.5 and 235.2 m^2^ g^−1^, respectively. Due to the large surface area, these samples can absorb more methylene blue.

As shown in Supplementary Figure S4, the UV–Vis absorbance spectra of the Fe-Ti/SF, Fe/SF and Ti/SF composites were evaluated. The adsorption edges of the Fe-Ti/SF, Fe/SF and Ti/SF composites are 510, 540 and 400 nm, respectively. The Kubelka–Munk function is applied to determine the band gaps of Fe-Ti/SF, Fe/SF and Ti/SF composites. As shown in inset of the Supplementary Figure S2, the band gaps of the Fe-Ti/SF, Fe/SF and Ti/SF composites from the energy versus [F(R∞)*hv*]^n^ can be estimated. R∞ and F(R∞) are the limiting reflectance and Kubelka–Munk function, respectively. The slope of the Tauc plot fit well at n = 1/2, which indicated an indirect transition. The band gaps of the Fe-Ti/SF, Fe/SF and Ti/SF composites are 2.23, 1.98 and 3.0 eV, respectively^30,31^. The partial absorption in the visible range is determined by the band gap energy value, which shows that these samples have potential photocatalytic activity.

**Figure 6:**
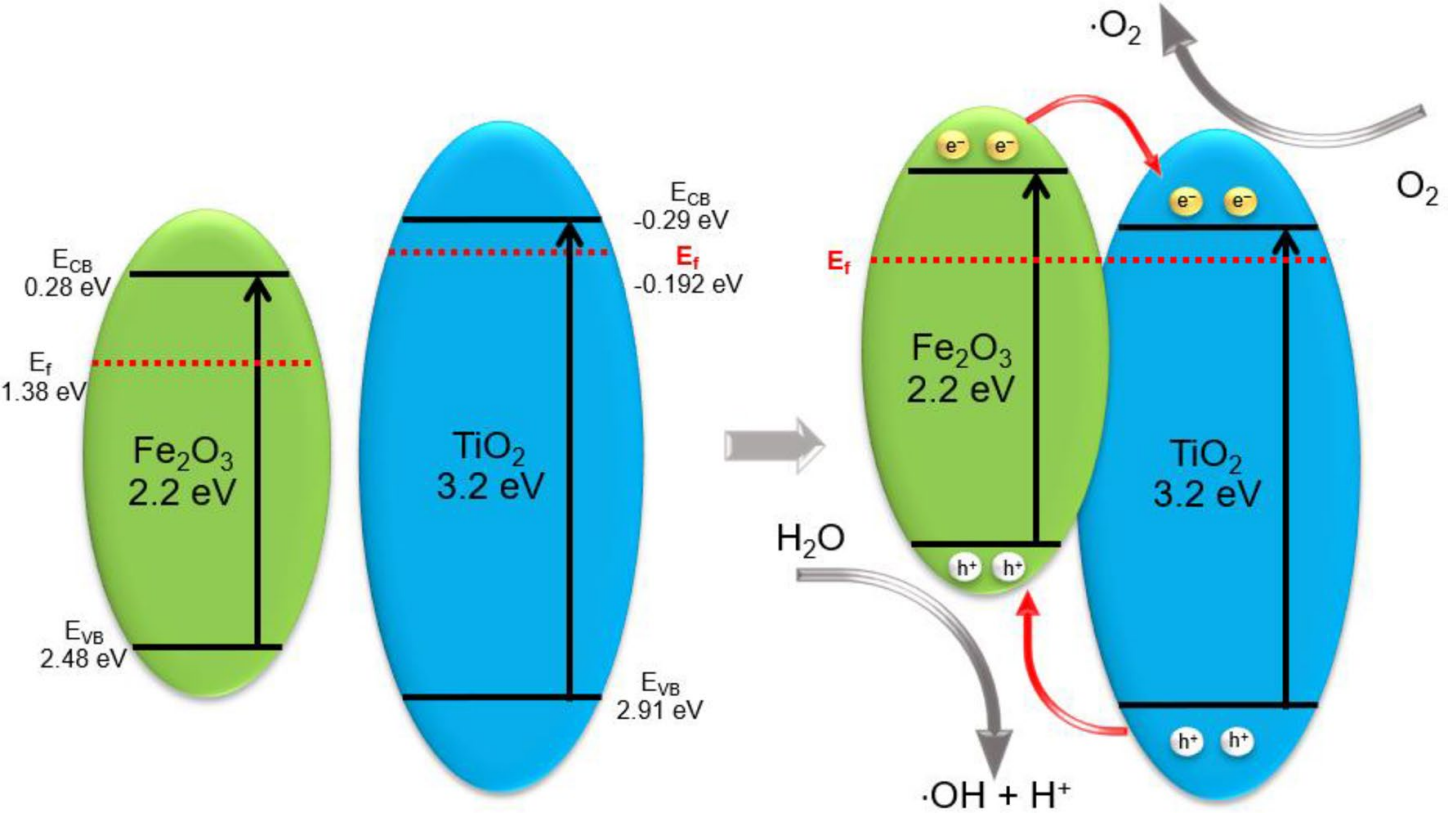
Schematic diagram of the band position of Fe-Ti/SF.

The schematic diagram of the band position of Fe-Ti/SF is shown in Fig. 6. The interparticle electrons intensity of Fe-Ti/SF is higher than the single component Fe_2_O_3_ or TiO_2_, which can promote the separation of electrons and holes. The Fermi levels of the Fe_2_O_3_ and TiO_2_ are lower than their conduction band (CB). In the heterostructure, the Fermi levels of Fe_2_O_3_ and TiO_2_ reach a new equalization state. There is a new electric field between the Fe_2_O_3_ and TiO_2_. The interparticle electrons rapidly transfer in the heterostructure between the Fe_2_O_3_ and TiO_2_. The electrons of a higher CB transfer to the lower one, the holes move in the opposite way. The electrons of Fe_2_O_3_ (CB) transfer to TiO_2_ (CB). The holes of TiO_2_ (VB) transfer to Fe_2_O_3_ (VB). The electrons are trapped by O_2_ (dissolved in the methylene blue) to form ^⋅^O_2_, the ^⋅^O_2_ oxidized the methylene blue. Finally, the methylene blue is degraded in CO_2_ and H_2_O.

**Figure 7:**
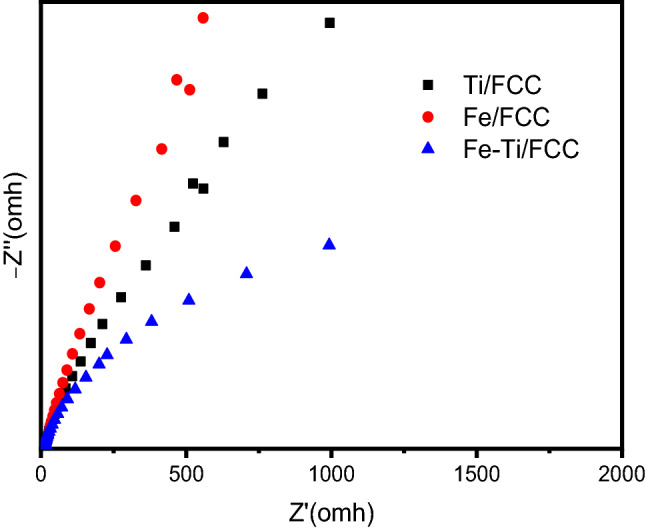
Electrochemical impedance spectroscopy (EIS) analysis of Fe-Ti/SF, Fe/SF and Ti/SF.

The electron transfer efficiency can be measured by the electrochemical impedance spectroscopy (EIS). As shown in Fig. 7, the radius (Nyquist plot) of the Fe-Ti/SF is much smaller than those of Fe/SF and Ti/SF, which shows that Fe-Ti/SF has lower electron transfer resistance. The heterostructure of the Fe_2_O_3_ and TiO_2_ enhances the separation of the electrons and holes.

**Figure 8:**
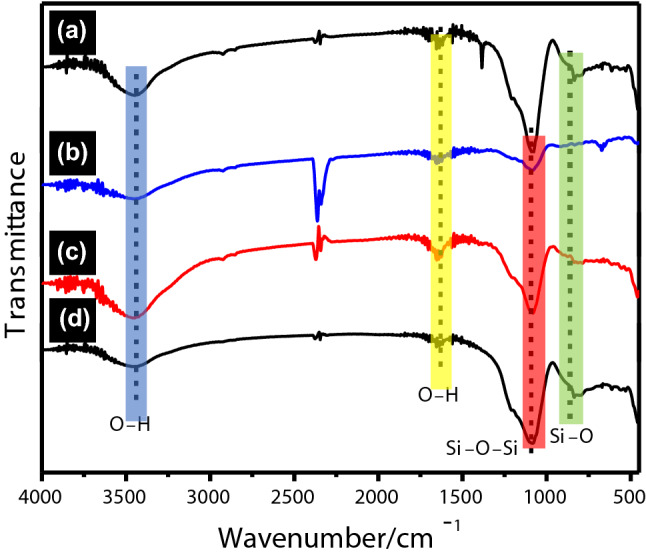
FT-IR spectra of (**a**) spent FCC catalyst, (**b**) Fe/SF, (**c**) Fe-Ti/SF and (**d**) Ti/SF, respectively.

In Fig. 8, the Fourier transform infrared spectroscopy (FT-IR) spectra of the spent FCC catalyst, Fe/SF, Fe-Ti/SF and Ti/SF are measured. The peak at 3,425 cm^−1^ corresponds to the absorption the stretching vibration of O‒H group stretching. The peak at 1645 cm^−1^ corresponds to the bending vibration absorption of the O‒H^32^. In Fig. 8a–d, the peak at 1,082 cm^−1^, 848 cm^−1^ and 456 cm^−1^ correspond to the stretching or bending vibration of the Si–O–Si^33,34^. In the samples of the (b) Fe/SF and (c) Fe-Ti/SF, the absorption peaks at 560, 610, 1,100, and 3,500 cm^−1^ correspond to the stretching vibration of Fe–O. In Fig. 8d, the peaks at 910 cm^−1^ to 960 cm^−1^ corresponds to the stretching vibration of the Ti‒O‒Si.

**Figure 9:**
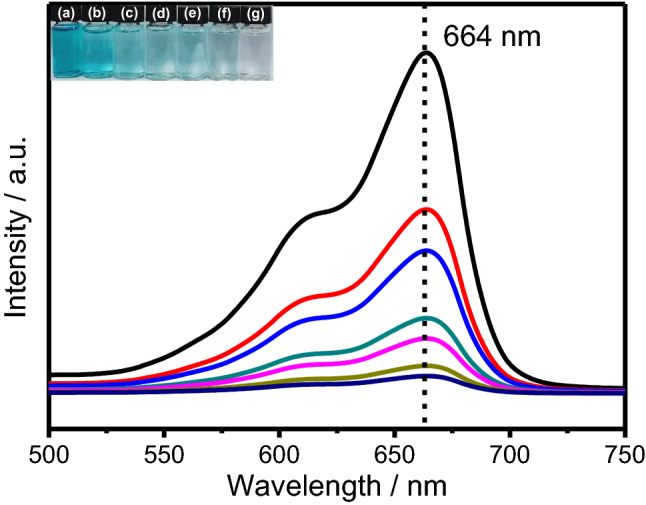
UV–Vis adsorption spectra of degradation of methylene blue with Fe-Ti/SF composite. Inset shows the photographs of methylene blue solution. The inset shows the photographs of methylene blue solution: the 0 min, 20 min, 40 min, 60 min, 80 min, 100 min and 120 min, which corresponds with (**a**–**g**), respectively.

In Fig. 9, the photocatalytic performance of the Fe-Ti/SF is evaluated by the methylene blue degradation experiment. With increase of the reaction time, the methylene blue characteristic peak is gradually declined, which indicates concentration of methylene blue is gradually declined. The photocatalytic degradation efficiency of the methylene blue is ~ 94.2%. The inset of Fig. 9 shows the color change of the methylene blue. Figure. S5 shows the degradation of methylene blue with the Fe-Ti/SF, Fe/SF and Ti/SF composites. The methylene blue degradation efficiencies (with Fe-Ti/SF, Fe/SF and Ti/SF composites) are ~ 94.2%, ~ 22.3% and ~ 54.0% in 120 min, respectively. The results show that the Fe-Ti/SF composite has the highest photocatalytic activity. Supplementary Figure S6 shows the photocatalytic degradation efficiency of methylene blue with Fe-Ti/SF, Fe_2_O_3_-TiO_2_, Fe_2_O_3_ and TiO_2_.

**Figure 10:**
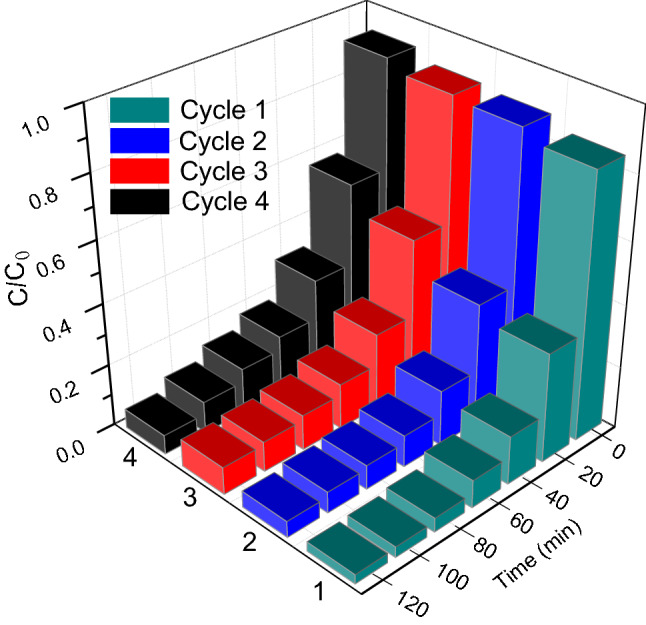
Recycling experiments of photocatalytic reduction of methylene blue with Fe-Ti/SF.

As shown in Fig. 10, the recycling experiments of the Fe-Ti/SF composite are implemented by the methylene blue degradation, which evaluates the stability of this Fe-Ti/SF composite. After the fourth cycle reaction, the photocatalytic activity of the Fe-Ti/SF composite is not significant loss. The degradation efficiency of the methylene blue with Fe-Ti/SF composite decreases to 84.0% from the pristine degradation efficiency (94.2%). These results indicate that the Fe-Ti/SF exhibit a relatively stable photocatalytic performance.

## Conclusion

In summary, the Fe-Ti/SF, Fe/SF and Ti/SF composites have been successfully fabricated via a modified-impregnation method. The adsorption and degradation tests were carried out for 120 min to evaluate Fe-Ti/SF, Fe/SF and Ti/SF photocatalytic performance, the methylene blue degradation efficiencies are ~ 94.2%, ~ 22.3% and ~ 54.0%, respectively. The interparticle electrons rapidly transfer in the heterostructure between the Fe_2_O_3_ and TiO_2_. The radius (Nyquist plot) of the Fe-Ti/SF is much smaller than those of Fe/SF and Ti/SF, which shows Fe-Ti/SF has lower electron transfer resistance and fast charge transfer rate. After the fourth cycle reaction, the photocatalytic activity of the Fe-Ti/SF composite reduces 10.2% (the photocatalytic degradation efficiency of methylene blue is from 94.2 to 84.0%). The spent FCC catalyst loaded with Fe_2_O_3_ and TiO_2_ has stable photocatalytic performance. This modified-impregnation method, the spent FCC catalyst is used as the photocatalyst supports, which provides a potential method to dispose the spent FCC catalyst in the areas of environment protection.

## Supplementary information

Supplementary file1

## References

[1] Shi J (2016). Nitrogen chemistry and coke transformation of FCC coked catalyst during the regeneration process. Sci. Rep..

[2] Ferella F (2019). Synthesis of zeolites from spent fluid catalytic cracking catalyst. J. Clean. Prod..

[3] Vogt ETC, Weckhuysen BM (2015). Fluid catalytic cracking: Recent developments on the grand old lady of zeolite catalysis. Chem. Soc. Rev..

[4] Yuan L (2019). Adsorption and mechanistic study for phosphate removal by magnetic Fe_3_O_4_-doped spent FCC catalysts adsorbent. Chemosphere.

[5] Chen X (2019). Synthesis, characterization and activity performance of nickel-loaded spent FCC catalyst for pine gum hydrogenation. RSC Adv..

[6] Le T, Wang Q, Ravindra AV, Li X, Ju S (2019). Microwave intensified synthesis of zeolite-Y from spent FCC catalyst after acid activation. J. Alloys Compd..

[7] Wu L, Khalil F, Smith GM, Yilmaz B, McGuire R (2015). Effect of solvent on the impregnation of contaminant nickel for laboratory deactivation of FCC catalysts. Micropor. Mesopor. Mat..

[8] Chen CM, Yu J, Yoza BA, Li QX, Wang G (2015). A novel “wastes-treat-wastes” technology: Role and potential of spent fluid catalytic cracking catalyst assisted ozonation of petrochemical wastewater. J. Environ. Manag..

[9] Akcil A, Vegliò F, Ferella F, Okudan MD, Tuncuk A (2015). A review of metal recovery from spent petroleum catalysts and ash. Waste Manag..

[10] Su B, Shi L, Liu N, Wang X, Meng X (2019). Removal of sulfur compounds from LPG by heteropoly acid-modified spent FCC catalyst. Appl. Organomet. Chen..

[11] Trivedi PA, Solanki NM, Butani N, Parikh PA (2014). Investigation on corrosion control of mild steel buried in soil by spent FCC catalyst coating. J. Ind. Eng. Chem..

[12] Le-Phuc N (2018). Towards efficient extraction of La(III) from spent FCC catalysts by alkaline pre-treatment. Miner. Eng..

[13] Neves R, Vicente C, Castela A, Montemor MF (2015). Durability performance of concrete incorporating spent fluid cracking catalyst. Cement. Concret. Comp..

[14] Scherzer J (1989). Octane-enhancing, zeolitic FCC catalysts: Scientific and technical aspects. Catal. Rev..

[15] Trivedi PA, Parmar PR, Parikh PA (2014). Spent FCC catalyst: Potential anti-corrosive and anti-biofouling material. J. Ind. Eng. Chem..

[16] Wang T (2019). Preparation of ordered TiO_2_ nanofibers/nanotubes by magnetic field assisted electrospinning and the study of their photocatalytic properties. Ceram. Int..

[17] Mohammadi M, Rezaee Roknabadi M, Behdani M, Kompany A (2019). Enhancement of visible and UV light photocatalytic activity of rGO-TiO_2_ nanocomposites: The effect of TiO_2_/graphene oxide weight ratio. Ceram. Int..

[18] Zhao Y (2019). Tuning oxygen vacancies in ultrathin TiO_2_ nanosheets to boost photocatalytic nitrogen fixation up to 700 nm. Adv. Mater..

[19] Li X, Qian JH, Xu JS, Xing JJET (2018). Synthesis, characterization and electrical properties of TiO_2_ modified with SiO_2_ and antimony-doped tin oxide. J. Mater. Sci. Mater. Electron..

[20] Wang W (2019). Edge-enriched ultrathin MoS_2_ embedded yolk-shell TiO_2_ with boosted charge transfer for superior photocatalytic H_2_ evolution. Adv. Funct. Mater..

[21] Low J, Dai B, Tong T, Jiang C, Yu J (2019). In situ irradiated X-ray photoelectron spectroscopy investigation on a direct Z-scheme TiO_2_/CdS composite film photocatalyst. Adv. Mater..

[22] Shirai K (2018). Water-assisted hole trapping at the highly curved surface of nano-TiO_2_ photocatalyst. J. Am. Chem. Soc..

[23] Chen Q, Zhou M, Zhang ZM, Tang T, Wang T (2017). Preparation of TiO_2_ nanotubes/reduced graphene oxide binary nanocomposites enhanced photocatalytic properties. J. Mater. Sci. Mater. Electron..

[24] Perveen S, Farrukh MA (2017). Influence of lanthanum precursors on the heterogeneous La/ SnO_2_–TiO_2_ nanocatalyst with enhanced catalytic activity under visible light. J. Mater. Sci. Mater. Electron..

[25] Ilkhechi NN, Ghobadi N, Akbarpour MR (2017). Enhanced optical and photo catalytic properties of V and La co doped TiO_2_ nanoparticles. J. Mater. Sci. Mater. Electron..

[26] de Krafft KE, Wang C, Lin W (2012). Metal-organic framework templated synthesis of Fe_2_O_3_/TiO_2_ nanocomposite for hydrogen production. Adv. Mater. Res..

[27] Silvestri S, Foletto EL (2017). Preparation and characterization of Fe_2_O_3_/TiO_2_/clay plates and their use as photocatalysts. Ceram. Int..

[28] Davari N, Farhadian M, Nazar ARS, Homayoonfal M (2017). Degradation of diphenhydramine by the photocatalysts of ZnO/Fe_2_O_3_ and TiO_2_/Fe_2_O_3_ based on clinoptilolite: Structural and operational comparison. J. Environ. Chem. Eng..

[29] Eskandari P, Farhadian M, Solaimany Nazar AR, Jeon B-H (2019). Adsorption and photodegradation efficiency of TiO_2_/Fe_2_O_3_/PAC and TiO_2_/Fe_2_O_3_/zeolite nanophotocatalysts for the removal of cyanide. Ind. Eng. Chem. Res..

[30] Xu JS, Pan CS, Takata T, Domen K (2015). Photocatalytic overall water splitting on the perovskite-type transition metal oxynitride CaTaO_2_N under visible light irradiation. Chem. Commun..

[31] Zhang HD (2014). Microwave-assisted synthesis of Cu_2_O microcrystals with systematic shape evolution from octahedral to cubic and their comparative photocatalytic activities. RSC Adv..

[32] Yosefi L, Haghighi M (2018). Fabrication of nanostructured flowerlike p-BiOI/p-NiO heterostructure and its efficient photocatalytic performance in water treatment under visible-light irradiation. Appl. Catal. B Environ..

[33] Król M, Minkiewicz J, Mozgawa W (2016). IR spectroscopy studies of zeolites in geopolymeric materials derived from kaolinite. J. Mol. Struct..

[34] Tang R, Chen T, Chen Y, Zhang Y, Wang G (2014). Core-shell TiO_2_@SiO_2_ catalyst for transesterification of dimethyl carbonate and phenol to diphenyl carbonate. Chin. J. Catal..

