# Supplementary material for: Photocatalytic degradation of methylene blue with spent FCC catalyst loaded with ferric oxide and titanium dioxide
Source: Sci Rep. 2020 Jul 29;10:12730. doi: 10.1038/s41598-020-69643-2 (PMC7391759; doi:10.1038/s41598-020-69643-2)
Supplement: Supplementary file 1 — Supplementary file1 [file 41598_2020_69643_MOESM1_ESM.docx]

**Photocatalytic Degradation of Methylene Blue Dye by Spent FCC Catalyst Loaded with Ferric Oxide and Titanium Dioxide**

Jiasheng Xu*^1,2^*, Te Zhang*^2^*, Jie Zhang*^1*^*

*^1^College of Chemistry, Chemical Engineering and Environmental Engineering, Liaoning Shihua University, Fushun, 113001, P.R. China.*

*^2^Liaoning Province Key Laboratory for Synthesis and Application of Functional Compounds, College of Chemistry and Chemical Engineering, Bohai University, Jinzhou 121013, P.R. China.*

**Correspondence and requests for materials should be addressed to J. Z. (email:* [*jiezhang@lnpu.edu.cn*](mailto:jiezhang@lnpu.edu.cn)*)*

**Supplementary material**

**(c)**

**(b)**

**(a)**


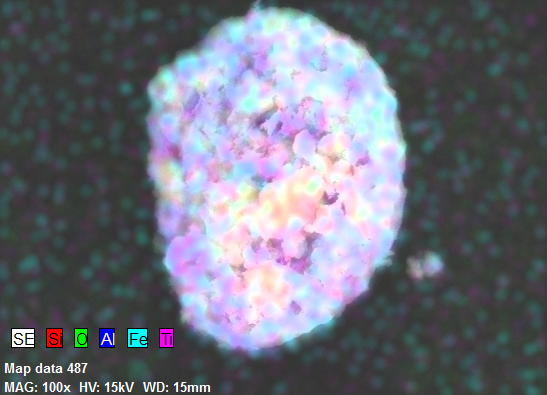

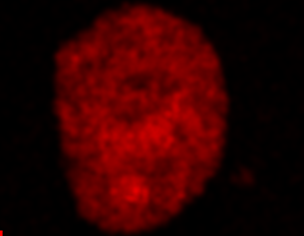

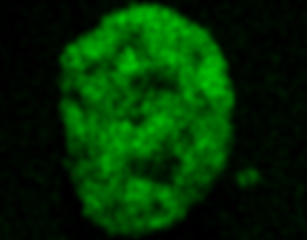

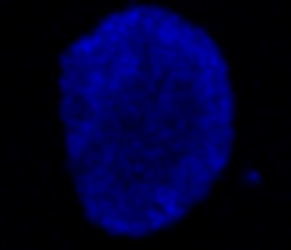

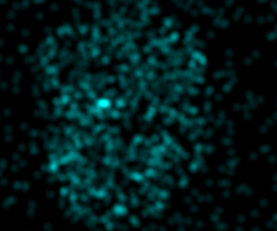

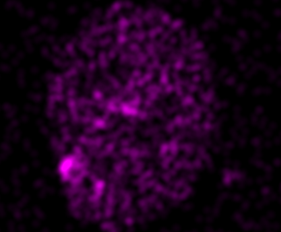


**(f)**

**(e)**

**(d)**

Figure. S1. Overlapped elemental mapping image of Si, O, Al, Fe and Ti, the energy dispersive spectroscopy (EDS) mapping of the selected area take from the green rectangular frame in (a) SEM image of Fe-Ti/SF, (b) Si mapping, (c) O mapping, (d) Al mapping, (e) Fe mapping and (f) Ti mapping.

Figure. S2. High-resolution XPS spectra of (a) Fe 2p, (b) Ti 2p, (c) Al 2p, (d) Si 2p and (e) O 1s for Fe-Ti/SF composite.


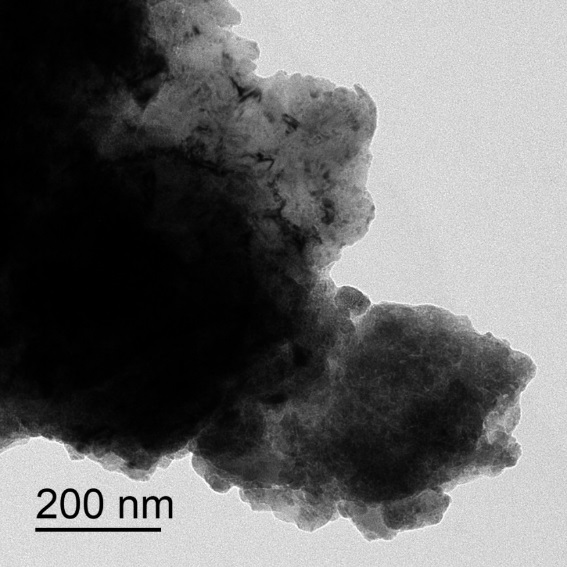

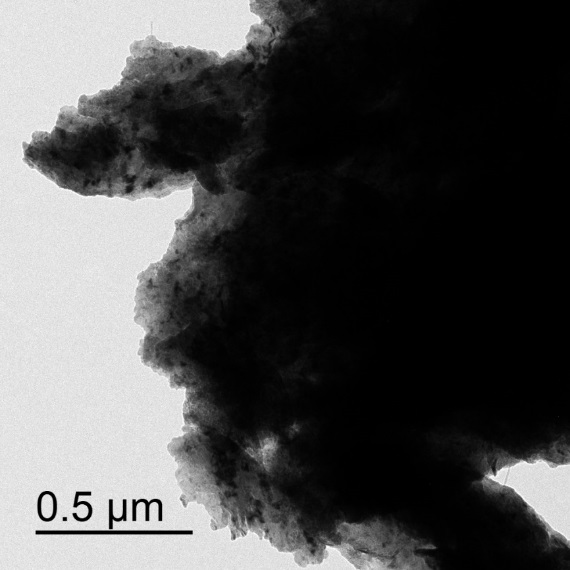


**(b)**

**(a)**


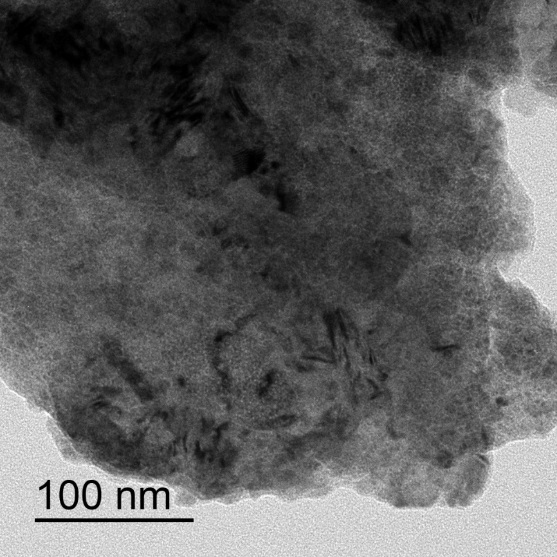


**(c)**

Figure. S3. (a-c) TEM images of the Fe-Ti/SF sample, scale bars = 0.5 μm, 200 nm and 100 nm, respectively.

**(b)**

**(a)**

**(c)**

Figure. S4. The UV-Vis reflection spectra of (a) Fe-Ti/SF, (b) Fe/SF and (c) Ti/SF samples were measured. The insets show the band gap transformations via Kubelka-Munk function.

Figure. S5. Concentration change of methylene blue in the same time interval with Fe-Ti/SF, Fe/SF and Ti/SF.

Figure. S6. The photocatalytic degradation efficiency of methylene blue with Fe-Ti/SF, Fe_2_O_3_-TiO_2_, Fe_2_O_3_ and TiO_2_.
